# Exploring the recurrence and metastasis of breast invasive ductal carcinoma based on machine learning and survival analysis

**DOI:** 10.3389/fonc.2026.1734379

**Published:** 2026-03-13

**Authors:** Aqiao Xu, Xiaobo Weng, Jing Zheng, Qian Cui, Gaoyan He, Yongran Cheng, Haitao Jiang, Mingzhu Wei, Shengjian Zhang

**Affiliations:** 1 Department of Radiology, The Central Hospital Affiliatedo Shaoxing University (Shaoxing Central Hospital), Shaoxing, China; 2 Department of Radiology, Shaoxing Maternity and Child Health Care Hospital, Shaoxing, China; 3 School of Public Health, Hangzhou Medical College, Hangzhou, China; 4 Department of Radiology, Cancer Hospital of the University of Chinese Academy of Sciences (Zhejiang Cancer Hospital), Hangzhou, China; 5 Department of Radiology, Shaoxing People’s Hospital (Zhejiang University Shaoxing Hospital), Shaoxing, China; 6 Department of Radiology, Fudan University Shanghai Cancer Center, Shanghai, China

**Keywords:** invasive ductal carcinoma, machine learning, metastasis, radiomics, recurrence-free survival

## Abstract

**Objective:**

Invasive ductal carcinoma (IDC), the predominant histopathological subtype comprising about 80% of breast malignancies, continues to pose a significant clinical challenge due to frequent recurrence. Existing relapse prediction models remain limited in accuracy and generalizability. This study aimed to construct and validate machine learning–based models for predicting 5-year (short- to medium term) recurrence and metastasis risk in IDC, based on recurrence-free survival (RFS) analysis.

**Methods:**

A total of 640 IDC cases diagnosed between January 2017 and December 2019 were enrolled, data were partitioned into three sets: the training set (n = 303) from Fudan University Shanghai Cancer Center; the validation set (n = 217) from Shaoxing Central Hospital; and the test set (n = 120) from Zhejiang Cancer Hospital. Independent prognostic factors were identified through univariate and multivariate Cox regression analyses. Three predictive strategies were implemented: evaluating recurrence risk, distinguishing local from distant recurrence, and identifying metastatic sites. Light Gradient Boosting Machine (LGBM), XGBoost (XGB), Random Forest (RF), k-Nearest Neighbor (KNN), Neural Network (NN), and Support Vector Machine (SVM) were trained and validated.

**Results:**

The median follow-up duration was 5.7 years. Multivariate Cox regression analyses identified multiple factors significantly associated with RFS, including the rad-score, Ki-67 index, lymph node metastasis, tumor histological grade, and breast cancer family history in first- or second-degree relatives (all *p* < 0.05). In contrast, age, menopausal status, and molecular subtype showed no significant association with recurrence risk in this cohort (*p =* 0.987, *p =* 0.987, and *p =* 0.960, respectively). The clinical-radiomic nomogram demonstrated strong in predictive IDC recurrence. The XGBoost model demonstrated robust and consistent predictive performance across all cohorts, achieving AUCs of 0.842, 0.848, and 0.912 on the training, validation, and test sets, respectively. On the independent test set, the model attained an accuracy of 93.8%, sensitivity of 96.3%, and specificity of 79.6%.Furthermore, density plots of the radiomic score and Ki-67 index effectively differentiated between local recurrence, bone metastasis, and metastases to other organs. Patients with lymph node metastasis and high histological grade demonstrated a higher frequency of metastases to distant organs, accounting for most cases and emphasizing the contrast with local recurrence and bone metastasis. Patients with a breast cancer family history displayed a distinct pattern of bone metastasis.

**Conclusion:**

This study underscores the utility of machine learning models in forecasting recurrence and metastatic behavior in IDC. The clinical-radiomic nomograms proved valuable for individualized surgical and therapeutic decision-making in IDC patients.

## 1 Introduction

Breast cancer remains the foremost cause of cancer-related deaths among women globally, with invasive ductal carcinoma (IDC) accounting for over 1.8 million new cases annually and demonstrating disproportionately aggressive recurrence behavior (1). Triple-negative breast cancer carries a higher risk of distant recurrence and death within five years, highlighting the limitations of current prognostic models (2, 3). The TNM staging system does not adequately address molecular heterogeneity, and genomic assays provide only moderate recurrence discrimination in node-positive breast cancer (C-index = 0.56–0.63) in multicenter validations (4–6). This prognostic insufficiency has tangible clinical implications: population studies reveal that excessive surveillance imaging in low-risk cohorts leads to unnecessary resource utilization, whereas high-risk patients often remain undetected under current protocols (7, 8). In this context, early relapse identification could guide targeted personalized therapy and improve prognostic prediction.

Recent studies have advanced the field of breast cancer prognosis. Jung et al. (9) developed predictive algorithms with 79.2% positive predictive value and 94.2% sensitivity using predictive algorithms validated by Kaplan–Meier (K-M) and Cox analyses for RFS, highlighting human epidermal growth factor receptor 2(HER2) status, clinical stage, and surgical intervention as key variables. the integration of multimodal imaging data and radiomics has become an important direction in improving survival prediction. Sheng et al. (10) developed a nomogram incorporating clinicopathological, ultrasonographic, and mammographic parameters, which achieved C-indices of 0.706 and 0.725 for predicting 3-year disease-free survival in training and validation cohorts, respectively. Similarly, Zhang et al. (11) integrated seven data modalities to predict relapse in HR+/HER2– breast cancer patients, attaining concordance indices (C-indices) of 0.871 and 0.869 on the training and testing sets, respectively. Furthermore, emerging imaging approaches and advanced AI-driven analyses, such as hyperspectral imaging and multimodal data integration, are pushing the boundaries of breast cancer diagnosis and risk evaluation (11, 12), while investigations of familial and socioeconomic determinants highlight the multifaceted nature of breast cancer risk (13).

Despite major progress in breast imaging technologies, converting computational advances into clinically applicable tools remains difficult, particularly in enabling the systematic and in-depth use of established imaging modalities. As a central method for breast cancer staging, dynamic contrast-enhanced magnetic resonance imaging (DCE-MRI) provides exceptional soft tissue contrast for assessing tumor extent and underlying tumor biology. Radiomic characterization of DCE-MRI is proposed to hold considerable, yet still underutilized, prognostic potential. However, existing radiomics studies largely emphasize binary recurrence prediction, with limited attention to organ-specific metastatic patterns, which are critical for tailoring personalized surveillance strategies. In addition, combining these quantitative imaging biomarkers with comprehensive clinicopathological profiles within a robust, externally validated machine learning framework remains insufficiently explored, thereby restricting clinical translation and real-world utility.

To address these gaps, the present study employed survival analysis and machine learning to construct predictive models elucidating the timing and determinants of IDC recurrence. Three analytic frameworks were designed: (i) evaluating recurrence risk using survival analysis; (ii) distinguishing local from distant metastasis; and (iii) predicting specific metastatic sites. By comprehensively integrating clinical and radiomic data, the study ensured high predictive discriminatory performance and clinical applicability.

## 2 Materials and methods

### 2.1 Patient data

From January 2017 to December 2019, we retrospectively collected breast tumor cases confirmed by clinical examination and diagnosed by ultrasound examination in this study. The inclusion criteria were as follows: (1)a histological diagnosis of invasive ductal carcinoma, no special type (IDC-NST);(2) no prior history of breast cancer or other malignancies; (3) no history of surgery, chemoradiotherapy, targeted therapy, or hormone therapy before enrollment; (4) Availability of preoperative breast DCE-MRI with acceptable quality; (5) Complete clinicopathological records; and (6) a minimum clinical follow-up period of 60 months. The exclusion criteria were: (1) contraindications to magnetic resonance imaging (MRI); (2) pregnancy or lactation, or a plan for pregnancy within six months; and (3) breast prosthesis implantation or severe imaging artifacts affecting MRI evaluation. (4) Incomplete follow-up data.

A total of 640 patients from three independent institutions met these criteria. The training set consisted of 303 consecutive patients from Fudan University Shanghai Cancer Center. The external validation set comprised 217 consecutive patients from Shaoxing Central Hospital. The test set included 120 consecutive patients from Zhejiang Cancer Hospital. This cohort allocation was designed to rigorously evaluate the model’s generalizability across diverse clinical settings.

Demographic and clinical information was retrieved from the electronic medical record systems of both institutions, including patient age, menopausal status, and breast cancer family history among first- and second-degree relatives. Pathological variables included tumor histological grade, progesterone receptor (PR) expression, HER2, Ki-67 index, estrogen receptor (ER), and lymph node metastasis (LNM).

**Figure 1:**
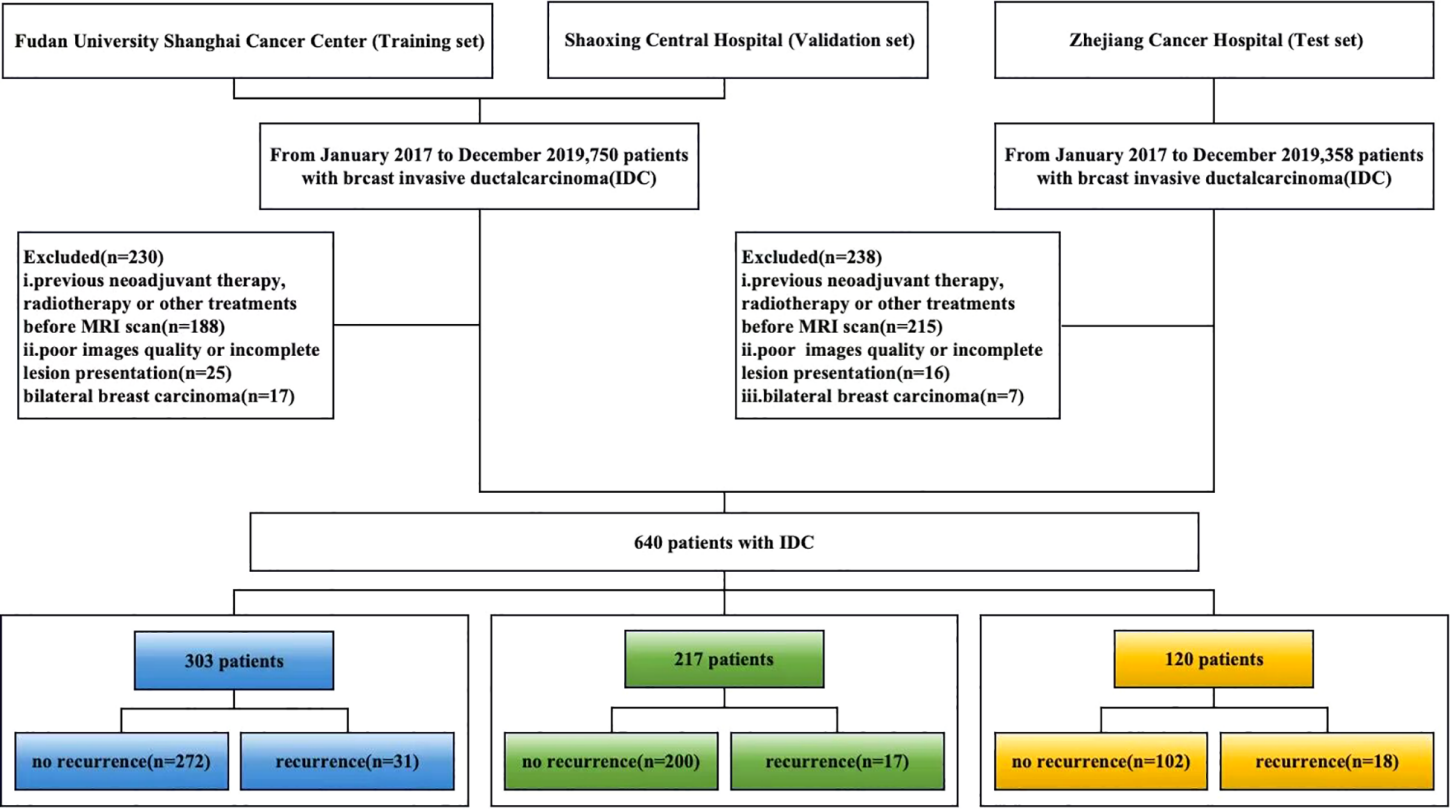
Patient selection workflow.

This study was approved by the institutional ethics committees of both participating centers, and the requirement for written informed consent was waived. The detailed workflow of patient recruitment and selection is presented in **Figure 1**.

### 2.2 Imaging examination

At Fudan University Shanghai Cancer Center, imaging was performed on an Aurora Dedicated Breast MRI System. A pre-contrast mask scan was obtained before the gadolinium diethylenetriamine penta-acetic acid (Gd-DTPA, 0.2 mmol kg^–1^) administration at a flow rate of 2.0 mL/s. Five consecutive contrast-enhanced phases were acquired, each lasting 120 s. The contrast protocol utilized T_1_-weighted sequence with fat and water suppression (TE = 29 ms, TR = 5 ms), with acquisition parameters of slice thickness = 1.1 mm, interslice spacing = 0 mm, field of view (FOV) = 360 mm×360 mm, and matrix = 360 × 128 × 360, yielding 160 slices per phase.

At Shaoxing Central Hospital, a 1.5 T Philips Achieva scanner (Philips Healthcare, Netherlands) equipped with a dedicated breast coil was used. The pre-contrast scan and contrast agent administration (Gd-DTPA, 0.2 mmol kg^–1^ at 2.0 mL/s) were identical to those used at the Shanghai center. Six contrast-enhanced phases were collected, each lasting 90 s. The contrast-enhanced sequence used T_1_-weighted scan with fat and water suppression (TE = 2.2 ms, TR = 5.0 ms), with a slice thickness = 1.0 mm, interslice spacing = 0.5 mm, matrix = 336 × 128 × 336, and FOV = 320 mm × 320 mm, resulting in 150 slices per phase.

At Zhejiang Cancer Hospital, a 3.0T Siemens Magnetom Verio A Tim System scanner (Siemens Healthineers, Germany) equipped with a 16-channel dedicated breast coil was used. A pre-contrast mask scan was obtained before the gadolinium-based contrast agent (Gd-DTPA, 0.2 mmol kg^–1^) administration at a flow rate of 3.0 mLmL/s. Six consecutive contrast-enhanced phases were acquired, each lasting 45 s. The contrast protocol used a FLASH 3D T1-weighted scan with fat and water suppression (TE = 3.93 ms, TR = 8 ms), with a slice thickness = 0.8 mm, interslice spacing = 0 mm, matrix = 448 × 72 × 448, FOV = 340 mm×340 mm, and yielding 128 slices per phase.

### 2.3 Images analysis

#### 2.3.1 Images preprocess

The original and transformed pre-therapy dynamic contrast-enhanced MRI (DCE-MRI) data were processed by semi-automatic delineation of three-dimensional regions of interest (3D ROIs), followed by manual correction on the DeepWise Scientific Research Platform v1.6 (http://keyan.deepwise.com/). The third post-contrast sequence of the dynamic enhancement series was used for analysis determined by each scanner specific DCE-MRI protocol, corresponding to about 240 s after contrast injection for the Aurora system and 180 s for the 1.5 T Philips Achieva scanner. At this enhancement phase, malignant tumors typically demonstrate maximal contrast relative to surrounding parenchyma, providing optimal conditions for accurate ROI definition and radiomic characterization.

Image Preprocessing and Normalization Prior to feature extraction, all DCE-MRI images underwent standardized preprocessing to ensure consistency and comparability across datasets. This included resampling to a uniform voxel size of 1.0×1.0×1.0 mm³ using B-spline interpolation, followed by intensity normalization through Z-score transformation based on the mean and standard deviation of the tumor voxel intensities within each patient. This step minimized scanner- and protocol-related variability and ensured that radiomic features reflected biological rather than technical differences.

#### 2.3.2 Feature extraction

Radiomic features were extracted from the segmented 3D ROIs using the open-source PyRadiomics library (version 3.0.1), compliant with the Imaging Biomarker Standardization Initiative (IBSI) guidelines. Features were derived from both original images and wavelet-transformed (eight frequency bands) images to capture multi-scale texture information. A total of 1,906 features were initially extracted from VOIs, including 396 first-order features, 14 shape features (including three 2D features), and 1496 texture features.

#### 2.3.3 Assessment of radiomic feature reproducibility and harmonization

Inter-observer reproducibility was assessed using intraclass correlation coefficients (ICC), and features with ICC< 0.75 were excluded. To mitigate potential scanner-related batch effects, ComBat harmonization was applied after feature extraction and before feature selection, using the training cohort as the reference batch. Learned parameters were subsequently transferred to the validation and test cohorts to avoid information leakage.

#### 2.3.4 Feature selection and rad-score construction

To address high dimensionality and multicollinearity, a two-step feature selection was performed exclusively on the training cohort:(1) Redundancy reduction: Spearman’s rank correlation analysis was applied, and one of any feature pair with correlation coefficient >0.9 was removed. (2) Predictive feature selection: The Least Absolute Shrinkage and Selection Operator (LASSO) (14, 15) regression with 10-fold cross-validation was used to identify the most informative features for recurrence prediction. The optimal penalty parameter ( λ) was determined by minimizing the binomial deviance.

The Rad-score for each patient was calculated as follows: $\text{Rad-score}=\sum_{i=1}^{13}w_{i}\cdot F_{i}$,where $w_{i}$ denotes the LASSO-derived coefficient for the selected feature $F_{i}$. The score was then standardized across the cohort for subsequent modeling.

#### 2.3.5 Model establishment and evaluation

Six machine learning classifiers—Light Gradient Boosting Machine (LGBM), XGBoost (XGB), Random Forest (RF), k-Nearest Neighbor (KNN), Neural Network (NN), and Support Vector Machine (SVM)—were evaluated for recurrence prediction (16, 17). All model development procedures were performed exclusively within the training cohort to avoid information leakage.

To address class imbalance, the Synthetic Minority Over-sampling Technique (SMOTE) was strategically applied exclusively during the training phase within each cross-validation folds. Model hyperparameters were optimized using a nested cross-validation framework, with an outer 5-fold cross-validation loop for perA total of 640 IDC patients (mean age 50.1 ± 10.3 years) were allocated into training (n=303), validation (n=217), and independent test (n=120) sets. Key baseline characteristics, including age, Ki-67 index, and family history, were balanced across cohorts (p>0.05), while expected heterogeneity was observed in molecular subtype and lymph node status distributions. Performance evaluation and an inner 5-fold loop for hyperparameter tuning via grid search, using the area under the receiver operating characteristic curve (AUC) as the optimization metric. The specific parameters can be found in **Supplementary Material 1**.

Model performance was summarized by aggregating results across outer cross-validation folds. The classifier achieving the highest average AUC (LGBM) was subsequently retrained on the entire training cohort using the optimal hyperparameters and further evaluated on the independent external test set for final validation and interpretation.

### 2.4 Construction and validation of the clinical-radiomic nomogram

A clinical-radiomic nomogram was developed to provide an individualized, visual tool for predicting recurrence-free survival (RFS) probabilities. The nomogram was constructed based on the final multivariate Cox proportional hazards model, which included the following independent prognostic factors: Rad-score, Ki-67 index, lymph node metastasis status, histological grade, and family history of breast cancer.

Nomogram performance was assessed in terms of calibration and discrimination. Internal calibration was evaluated on the training cohort using 1,000 bootstrap resamples, with calibration curves comparing predicted and observed RFS probabilities at 5 and 7 years. Predictive discrimination was quantified using Harrell’s concordance index (C-index) in both the training and validation cohorts.

### 2.5 Follow-up and endpoints

All patients were followed up through telephone interviews or outpatient visits every six months until December 2024. Treatment protocols followed contemporary national guidelines at each center during 2017-2019.The primary endpoint was breast cancer recurrence or death from any cause. Recurrence included local and distant events. Local recurrence referred to ipsilateral breast, chest wall, axillary, or regional lymph node recurrence. In contrast distant metastasis referred to tumor spread to organs such as the lungs, liver, brain, and other sites. Recurrence events were confirmed by a Standard imaging modalities comprise computed tomography (CT), magnetic resonance imaging (MRI), bone scans, positron emission tomography (PET), etc., with final validation by a multidisciplinary tumor board at each participating institution.

### 2.6 Statistical methods

All analyses were performed using R v3.6.1 (http://www.Rproject.org).Continuous variables with normal distributions, comparisons were made using Student’s t-test, while categorical data were assessed with the chi-square test. Non-normally distributed variables were analyzed using the Mann–Whitney U test. All tests were two-sided, and a P value< 0.05 denoted statistical significance.

Candidate prognostic variables for Cox regression were selected based on clinical relevance and prior literature, including age, menopausal status, histological grade, lymph node metastasis, Ki-67 index, molecular subtype, family history of breast cancer, and the Rad-score. Multicollinearity among variables was assessed using variance inflation factors (VIF), with all values below 3, indicating acceptable collinearity. Subsequently, multivariate Cox proportional hazards regression was used to identify independent prognostic factors for RFS. The model was constructed using a backward stepwise selection approach based on the Akaike Information Criterion (AIC). Proportional hazards assumptions were evaluated using Schoenfeld residuals and were satisfied for all variables. Hazard ratios (HRs) with 95% confidence intervals (CIs) were reported. Survival curves were generated using the Kaplan-Meier method and compared with the log-rank test. The optimal cutoff value for stratifying patients into high- and low-risk groups was determined using the survminer package.

Model performance was evaluated using multiple metrics. The discriminative ability of the radiomic signature and the machine learning classifiers was quantified using receiver operating characteristic (ROC) curve analysis and the area under the curve (AUC). For the nomogram, both discrimination and calibration were assessed. Discrimination was quantified using Harrell’s concordance index (C-index). Calibration, which evaluates the agreement between predicted probabilities and observed outcomes, was assessed visually with calibration curves and statistically using the Hosmer-Lemeshow test. Additionally, decision curve analysis (DCA) was conducted to evaluate the net clinical benefit.

## 3 Results

### 3.1 Recurrence-free survival analysis

**Figure 2:**
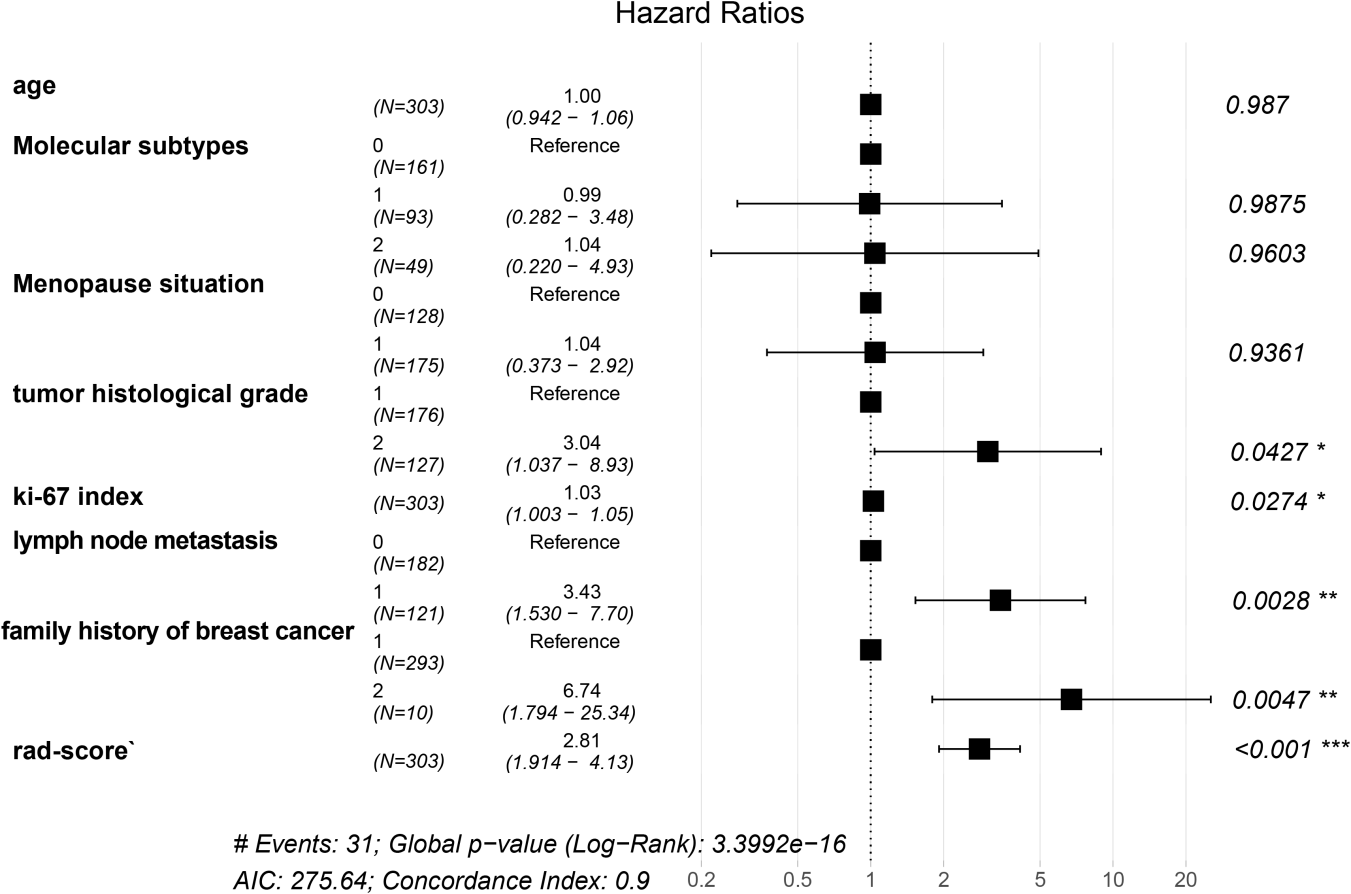
Multivariate cox regression analysis of RFS.

The mean age of patients was determined to be 50.07 ± 10.48 years, in a range of 16–86 years. A total of 640 IDC patients were allocated into training (n=303), validation (n=217), and independent test (n=120) sets. Key baseline characteristics, including age, Ki-67 index, and family history, were balanced across cohorts (p>0.05).The multivariate Cox regression analysis identified the following independent predictors for RFS: the Rad-score (HR = 2.81, 95%CI: 1.914–4.13, *p* < 0.001), family history of breast cancer (HR = 6.74, 95%CI: 1.794–25.34, *p* = 0.0028), lymph node metastasis (HR = 3.43, 95%CI: 1.530–7.70, *p* = 0.0028), tumor histological grade (HR = 3.04, 95%CI: 1.037–8.93, *p* = 0.0427), and Ki-67 index (HR = 1.03, 95%CI: 1.003–1.05, *p* = 0.0274). In contrast, age (HR = 1.00, 95%CI: 0.942–1.06, *p* = 0.987), molecular subtypes (HR = 1.04, 95%CI: 0.220–4.93, *p* = 0.9603), and menopausal status (HR = 1.04, 95%CI: 0.373–2.92, *p* = 0.9361) were not significantly associated with RFS in our study. Women with a family history of breast cancer had a higher recurrence risk compared with those without such a history. Tumor histological grade also played a significant role, with grade III (high-grade) tumors showing a higher risk of recurrence than grade I–II (low-grade) tumors. Furthermore, a high Ki-67 index and the presence of LNM were identified as major risk factors for recurrence (**Figure 2**).

**Figure 3:**
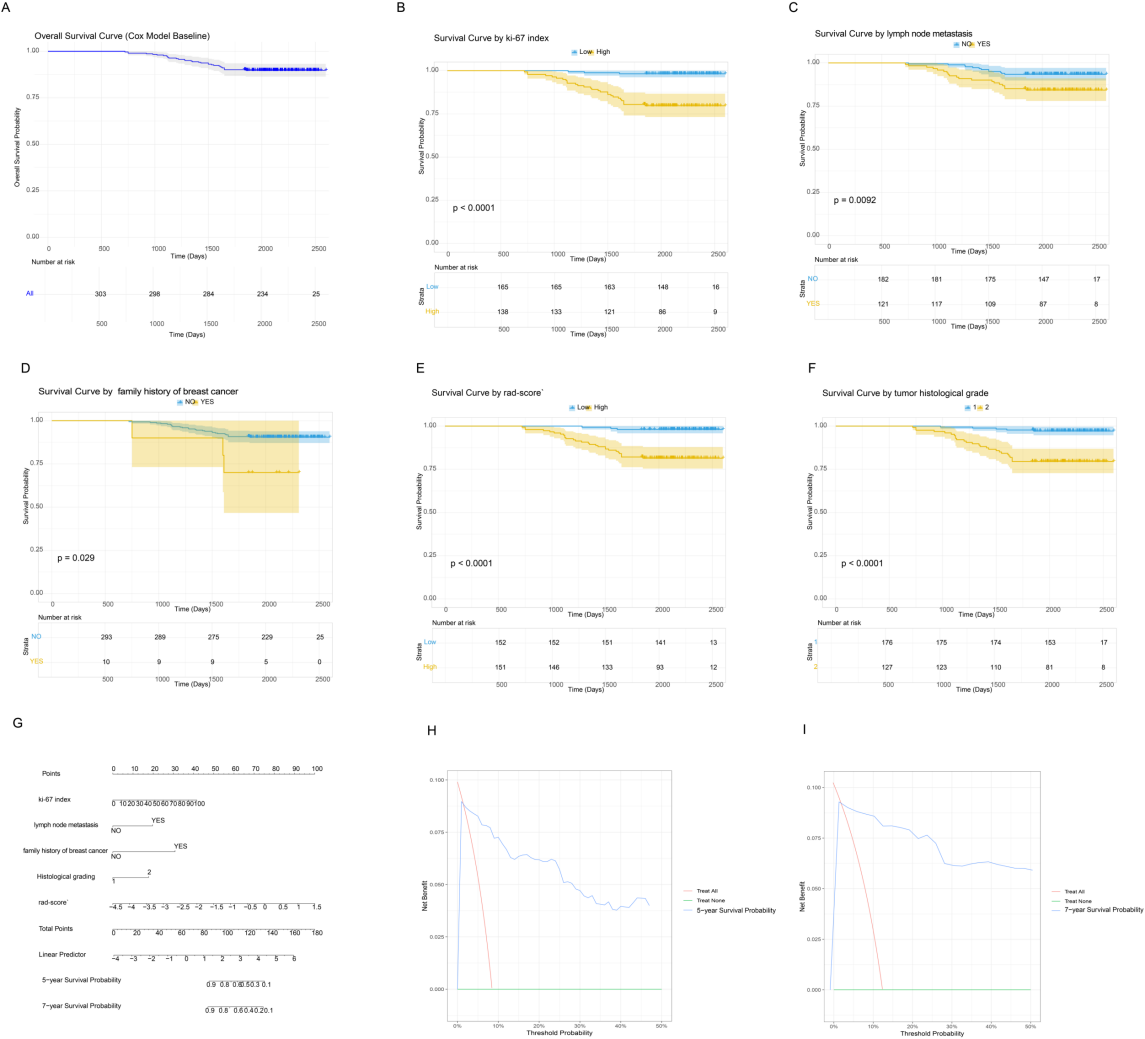
RFS analysis. **(A)** K–M RFS curve. K–M survival plots stratified by covariates: **(B)** Ki-67 index, **(C)** LNM, **(D)** breast cancer family history in first- and second-degree relatives, **(E)** Rad-score, **(F)** histological grade, **(G)** Nomogram for predicting RFS. **(H)** DCA for the nomogram predicting 5-year RFS. **(I)** DCA for the nomogram predicting 7-year RFS.

The K–M curve demonstrated an overall RFS rate of 0.872 (**Figure 3A**). K–M analyses for individual covariates were performed for visualization of survival curves (**Figures 3B–F**). The nomogram for predicting RFS (**Figure 3G**) exhibited strong predictive capability. The nomogram demonstrated good calibration in three cohorts. On the training set, the calibration curve for 5-year RFS prediction showed a slope of 0.96 (95% CI: 0.91–1.01) and an intercept of 0.02 (Hosmer-Lemeshow test, p = 0.32), indicating excellent agreement between predicted and observed outcomes (**Supplementary Figure 1A**). Similar good calibration was observed for 7-year RFS prediction. On the validation set, the calibration curve for 5-year RFS yielded a slope of 0.92 (95% CI: 0.85–0.99) and an intercept of 0.05 (Hosmer-Lemeshow test, *p* = 0.28) (**Supplementary Figure 1B**). The nomogram achieved C-indices of 0.82 (95% CI: 0.76–0.88) and 0.80 (95% CI: 0.73–0.87) On the training and validation sets, respectively, confirming its robust discriminative ability. And DCA results confirmed its clinical applicability for both 5-year and 7-year RFS prediction (**Figures 3H, I**). The DCA results demonstrate the clinical utility of the nomogram across a wide range of threshold probabilities (0.1 to 0.5). In clinical practice, the threshold probability reflects the clinician’s or patient’s willingness to accept the risks of intensified therapy in exchange for a potential reduction in recurrence risk. the use of our nomogram to guide decisions provides a net benefit of approximately 0.08 over both the “treat-all” and “treat-none” strategies. This translates to a net reduction of 8 unnecessary interventions per 100 patients without missing any patients who would benefit, compared to the best alternative strategy. This utility was consistent for both 5-year and 7-year RFS predictions, supporting its applicability in both medium- and long-term planning. And DCA results confirmed its clinical applicability for both 5-year and 7-year RFS prediction (**Figures 3H, I**, **Table 1**).

**Table 1:** Baseline data for the training set, validation set, and test set.

Characteristics	Classifications	Training set	Validation set	Test set	*p-value*
N		303	217	120	
Recurrence status (%)	no	272 (89.8)	200 (92.2)	102(85.0)	0.427
	yes	31 (10.2)	17 (7.8)	18(15.0)	
Age [mean (SD)]		50.5 (10.4)	50.0 (10.3)	48.95(9.8)	0.542
Molecular subtype (%)	Luminal	161 (53.1)	48 (22.1)	81(67.5)	0.13
	HER2-enriched	93 (30.7)	70 (32.3)	15(12.5)	
	Triple-negative	49 (16.2)	99 (45.6)	24(20.0)	
Menopausal status (%)	Post	128 (42.2)	134 (62.0)	79(65.8)	0.11
	Pre	175 (57.8)	82 (38.0)	41(34.2)	
Histological grades (%)	Low-grade	176 (58.1)	117 (53.9)	74(61.6)	0.367
	High-grade	127 (41.9)	100 (46.1)	46(38.4)	
Lymph node metastasis (%)	no	182 (60.1)	135 (62.2)	26(21.6)	0.547
	yes	121 (39.9)	82 (37.8)	94(78.4)	
Ki-67 index [mean (SD)]		35.6 (23.8)	33.8 (19.1)	37.65	0.148
Follow-up time [mean (SD)]		2130.5 (350.2)	2052.4 (407.6)	2214.65	0.05
Family history (%)	no	293 (96.7)	213 (98.2)	115(95.8)	0.454
	yes	10 (3.3)	4 (1.8)	5(4.2)	
Radiomics_score [mean (SD)]		-2.5 (0.9)	-3.4 (0.6)	-3.3(0.7)	0.537
RFS rate% (95%CI)					0.09
5 years recurrence-free survival		90.1%(86.7%–93.5%)	92.5%(89.1%–96.1%)	91.2%(89.3%–96.5%)	
7 years recurrence-free survival		89.7%(86.4%–93.2%)	91.9%(88.3%–95.7%)	90.3%(89.0%–94.8%)	

### 3.2 Comparison of forecasting methods

The predictive performance of six machine learning classifiers for RFS is summarized in **Table 2**. On the independent test set, XGBoost achieved the highest AUC (0.912), followed by NN (0.904) and RF (0.894). XGBoost demonstrated an accuracy of 93.8%, sensitivity of 96.3%, and specificity of 79.6%; NN achieved 91.2% accuracy, 96.3% sensitivity, and 76.7% specificity; RF attained 92.6% accuracy, 98.4% sensitivity, and 58.9% specificity. On the training set, LGBM exhibited the highest AUC (0.889) and the best F1-score (0.946), with accuracy of 91.1%, sensitivity of 96.2%, and specificity of 88.3%. XGBoost demonstrated a competitive training AUC (0.842) but markedly lower training specificity (49.6%). SVM maintained stable performance across both cohorts (training AUC = 0.832, test AUC = 0.828), with consistently high sensitivity.

**Table 2:** Performance of six machine learning classifiers for predicting RFS status on the training and independent test set.

Model	Accuracy		Sensitivity		Specificity		Recall		F1-score		AUC	
Set	Training	Test	Training	Test	Training	Test	Training	Test	Training	Test	Training	Test
KNN	0.875	0.917	0.970	0.971	0.594	0.604	0.843	0.971	0.836	0.956	0.632	0.736
RF	0.850	0.926	0.980	0.984	0.594	0.589	0.822	0.982	0.841	0.961	0.637	0.894
SVM	0.821	0.912	0.980	0.985	0.851	0.784	0.894	0.984	0.822	0.954	0.832	0.828
NN	0.836	0.912	0.960	0.963	0.652	0.767	0.901	0.961	0.853	0.953	0.656	0.904
LGBM	0.911	0.899	0.962	0.953	0.883	0.760	0.913	0.952	0.946	0.945	0.889	0.886
XGB	0.903	0.938	0.950	0.963	0.496	0.796	0.852	0.950	0.852	0.950	0.842	0.912

**Figure 4:**
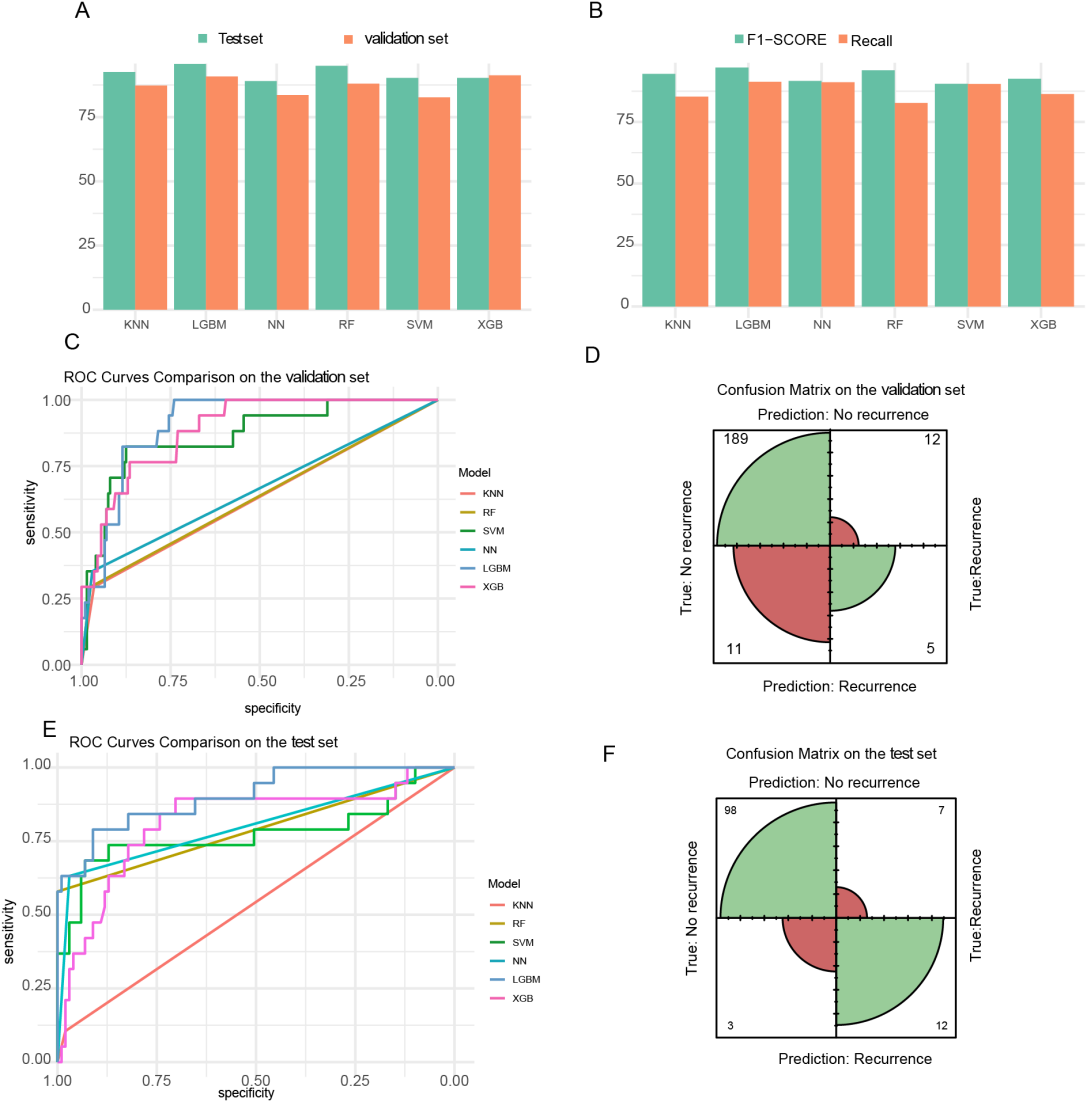
Recurrence vs. non-recurrence prediction results. **(A)** Comparison of validation set and test set accuracies across six machine learning models (KNN, LGBM, NN, RF, SVM, XGB). **(B)** Evaluation metrics (recall and F1−score) for the six models on the independent test set. **(C)** Combined ROC curves for all six models on the validation set. **(D)** Confusion matrix for predictions by the LGBM model on the validation set, displaying predicted classes (X−axis) versus true classes (Y−axis). True negatives (TN): 189, false negatives (FN): 12,false positives (FP): 11,true positives (TP):5. **(E)** Combined ROC curves for all six models on the independent test set **(F)** Confusion matrix for predictions by the LGBM model on the independent test set, showing predicted classes (X−axis) against true classes (Y−axis). True negatives (TN): 98, false negatives (FP): 7, false positives(FN):3,true positives (TP):12.

The accuracy of six machine learning models on the training and validation sets is compared, demonstrating that XGBoost and LGBM achieved the highest and most balanced performance across both sets (**Figure 4A**). In addition to accuracy, other performance metrics were evaluated, including recall and F1 score, as these are crucial for identifying positive recurrence cases that directly impact patient survival (**Figure 4B**). The combined receiver operating characteristic (ROC) curve for all six models on the validation set (**Figure 4C**) highlighted comparative performance. Misclassification biases were assessed using the confusion matrix for the LGBM model (**Figure 4D**). LGBM and XGBoost achieved the best predictive performance for IDC recurrence and metastasis, with the highest AUC values of 0.889 and 0.842, respectively (**Table 2**). No statistically significant differences were observed between their accuracies. We also conducted verification on the test set and obtained the similar results (**Figures 4E, F**),The models, particularly XGBoost, RF and LGBM exhibited stable and reliable performance metrics comparable to those on the training and validation set, with the AUC values of 0.899,0.869 and 0.849, respectively.

### 3.3 Data distribution insights

**Figure 5:**
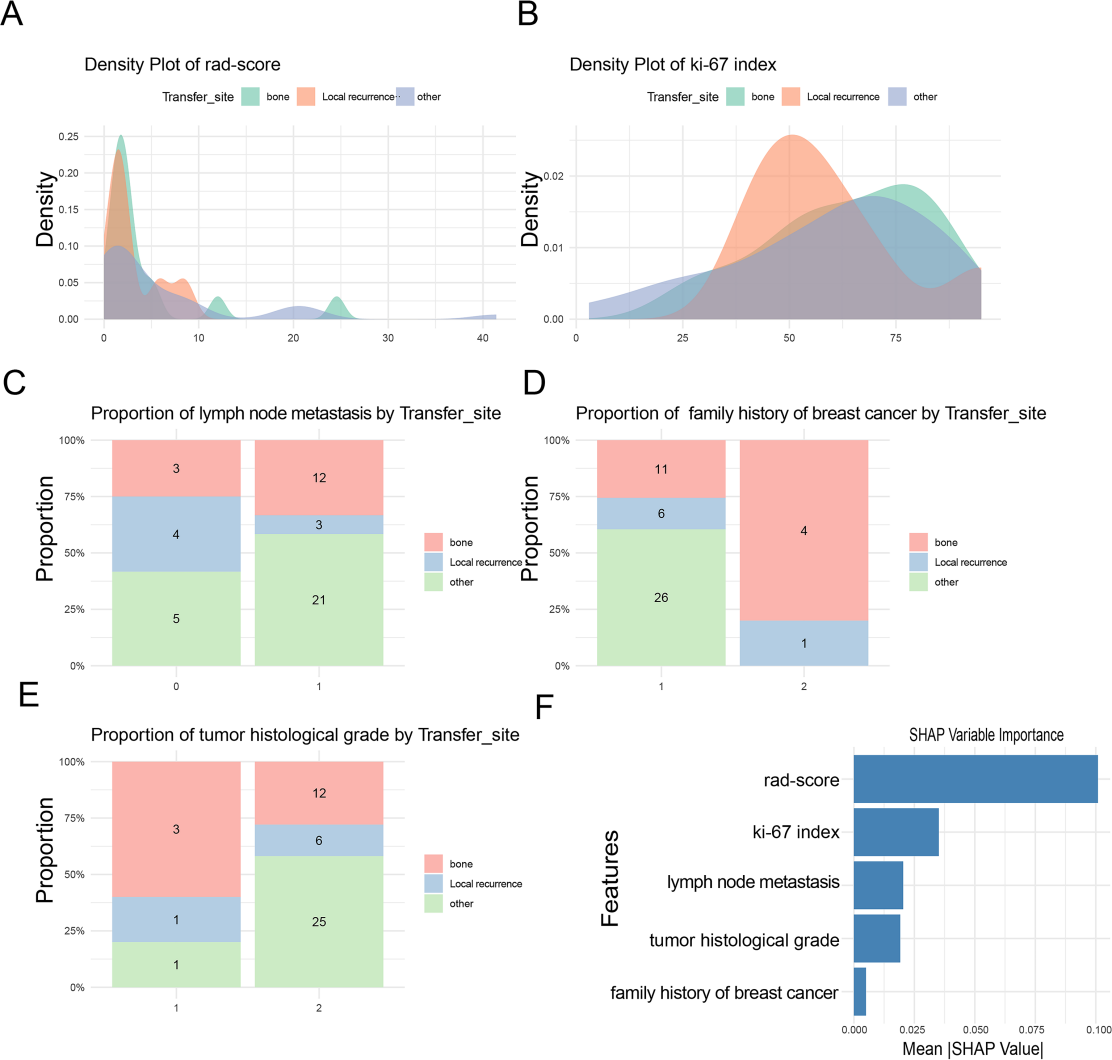
Feature distribution across metastatic locations. **(A)** Density plot of Rad-score showing distinct peaks for bone metastasis and local recurrence, and a lower peak for other organ metastases. **(B)** Density plot of Ki-67 index distinguishing local recurrence from distant metastasis. **(C)** LNM exhibits distinct distribution patterns from other organ metastases. **(D)** Breast cancer family history in first- and second-degree relatives showing a characteristic bone metastasis pattern. **(E)** High histological grade is associated with a distinct pattern of other organ metastasis. **(F)** SHAP feature importance plot.

The density plot of the radiomic score (**Figure 5A**) revealed a bimodal distribution. The first prominent peak corresponded to local recurrence and bone metastasis, while a lower secondary peak represented metastases to other organs, including the lungs, liver, brain, and omentum. The density plot of the Ki-67 index (**Figure 5B**) effectively distinguished local recurrence from distant metastasis, with local recurrence associated with a lower Ki-67 index.

On the feature distribution analysis (**Figures 5C–E**), each proportional chart represents the radiomic features distribution across different metastatic locations, providing insights into their biological variability. Patients without LNM and with low histological grade exhibited similar distribution patterns, suggesting minimal variation between these groups. Conversely, patients with LNM and high histological grade demonstrated a higher frequency of metastases to distant organs, accounting for most cases and emphasizing the contrast with local recurrence and bone metastasis. Patients with a breast cancer family history exhibited a distinct tendency toward bone metastasis.

The SHAP feature importance analysis for the RF model (**Figure 5F**) revealed that features such as Rad-score,Ki-67 index, LNM, histological grade, and family history were the most influential variables in predicting recurrence and metastasis.

**Figure 6:**
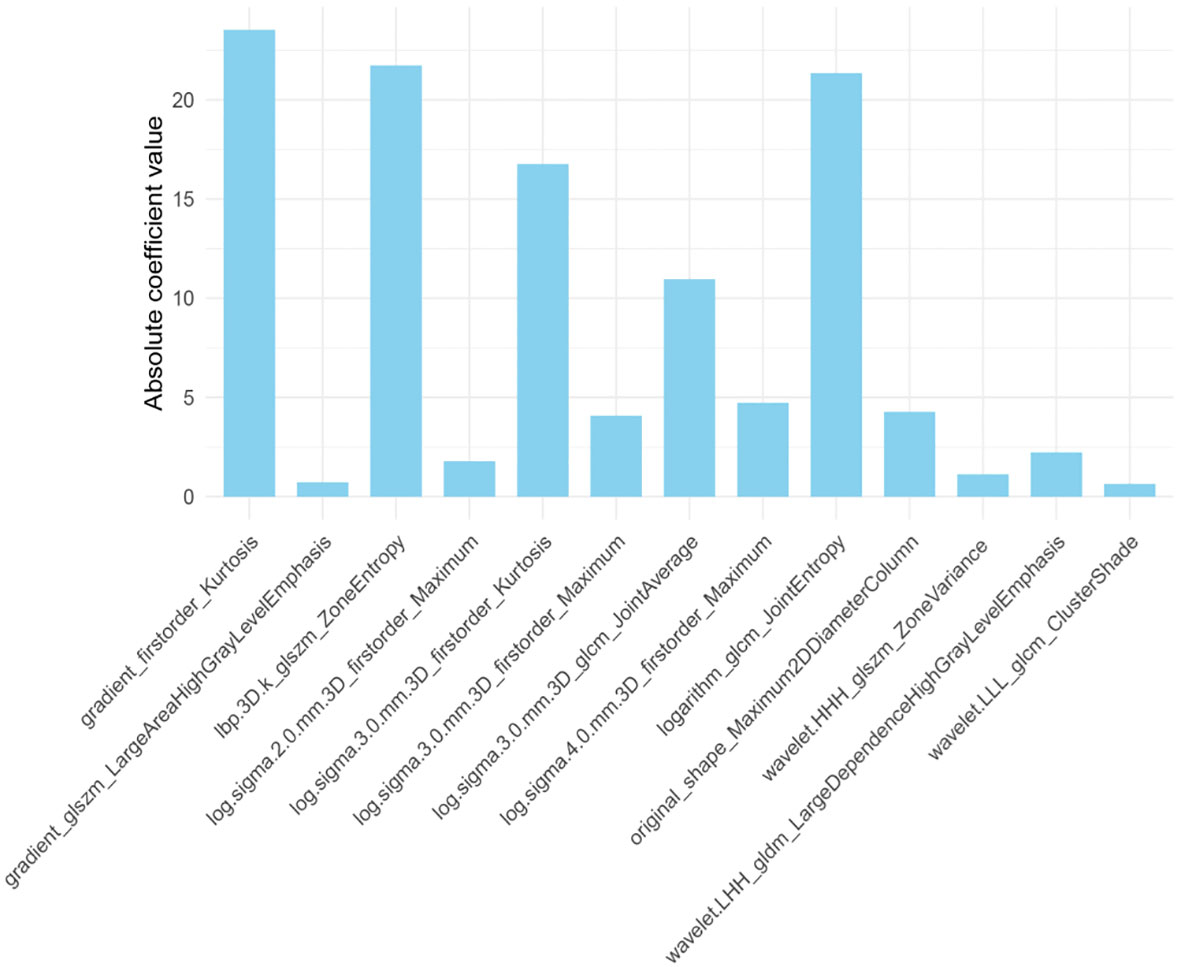
The 13 radiomic features with the highest discriminative value contributing to the Rad-score.

In the Rad-score analysis, 1906 radiomic features were initially extracted from the training set, comprising 396 first-order, 14 shape (including two-and three-dimensional metrics), and 1496 texture features. Ultimately, 13 optimal features were selected: 5 first-order, 3 gray-level co-occurrence matrix (GLCM), 3 gray-level size zone matrix (GLSZM), 1 gray-level dependence matrix (GLDM), and 1 two-dimensional shape feature. The 13 radiomic characteristics having the highest discriminative value contributing to the Rad-score are presented in **Figure 6**.

## 4 Discussion

The advent of artificial intelligence-based radiomics has enabled the identification of high- and low-risk patients with early-stage breast cancer and provided clinically valuable decision-support tools. This study developed and validated novel nomograms integrating radiomic, clinical, and pathological features for individualized prediction of recurrence and metastasis in IDC patients. Patients were stratified into high- and low-risk groups for RFS in advance, substantially improving predictive capability. The model effectively classified patients into distinct prognostic categories, enhancing prediction accuracy beyond conventional single-parameter systems. This approach holds significant potential for guiding individualized follow-up strategies. The LGBM model achieved an AUC of 0.889 on the test set for recurrence prediction, consistent with the findings of Noman et al. (18), who reported an AUC of 0.92 for the LGBM model in an external validation cohort. This performance significantly surpassed traditional prognostic benchmarks such as qualitative ultrasonographic evaluation (AUC = 0.72–0.78) (19) and the Nottingham Combined Histological Grade system (AUC = 0.65–0.70) (20). These results mark a substantial advancement over conventional diagnostic systems constrained by single-modality data inputs (21).

Survival analysis provided critical insights into the determinants of breast cancer recurrence. Multivariate Cox regression indicated that age and menopausal status were not statistically significant predictors of relapse, consistent with contemporary studies in which age-related factors are superseded by more robust prognostic indicators within multifactorial models (22, 23). Jung et al. (9)identified tumor grade, HER2 status, tumor size, and LNM as significant predictors, a pattern corroborated by the present study, wherein tumor histological grade, Ki-67 index, LNM, and breast cancer family history in first- and second-degree relatives emerged as significant variables in the multivariate model. This concordance underscores the consistency of these risk factors across independent cohorts. In the multivariate model, established prognostic factors such as molecular subtype did not retain independent statistical significance (*p* = 0.960). This finding may be attributed to several factors: First, the distribution of molecular subtypes differed between the training and validation sets (**Table 1**), potentially introducing cohort specific heterogeneity that diluted subtype-specific prognostic effects in the pooled analysis. Second, the prognostic information conveyed by molecular subtype may be partially captured by other correlated variables in the model, such as Ki-67 index (a key component in defining Luminal B vs Luminal A subtypes) histological grade and LNM. The inclusion of these more granular or continuous variables might have rendered the broader categorical variable of molecular subtype redundant in our specific model framework. Third, the classification strategy employed, which grouped Luminal A and Luminal B subtypes into a single category, may have obscured intrinsic prognostic differences between these entities. This phenomenon aligns with the known statistical principle that predictors appearing significant in univariate analyses may lose independent prognostic weight when modeled alongside collinear variables (24, 25).

In this analysis, 13 optimal features were ultimately retained for modeling, encompassing first-order, shape, GLDM, GLCM, and GLSZM characteristics—features primarily reflecting tumor heterogeneity. Among these, the two-dimensional maximum diameter (Shape_Maximum 2D diameter) represented tumor size, with tumor size identified as the most influential factor. This finding aligns with prior evidence linking larger tumors to increased risk of bone metastasis (26), reflecting the correlation between tumor dimension, biological aggressiveness, and poor prognosis. The prognostic value of kurtosis and entropy was also evident: higher values were associated with treatment failure, whereas lower values indicated a favorable therapeutic response, supporting their inclusion in the radiomic score for metastatic prediction (27, 28). Additional second- or higher-order features, such as zone variance, large-area high gray-level emphasis, joint average, large-dependence high gray-level emphasis, and cluster shade, were derived from both original and wavelet-transformed data. These metrics quantify textural irregularity and intra-tumoral heterogeneity, capturing subtle variations in gray-level patterns (29). Such parameters are increasingly recognized as essential for texture-based medical image analysis. Overall, combining literature-supported clinical risk factors with optimized radiomic features provides a robust and interpretable framework for enhancing breast cancer recurrence prediction.

The distinct peak observed in the distribution of radiomic scores and Ki-67 index across local recurrence, bone metastasis, and metastases to other sites indicates inherent differences in the biological and radiological features of metastatic lesions (30). This observation supports emerging evidence that radiomic features can quantify tumor microenvironmental heterogeneity and metastatic potential (31). The characteristic clustering of bone metastases likely reflects the unique osseous microenvironment that influences tumor phenotype and imaging appearance a concept substantiated by Tomography-based radiomics studies of bone metastasis detection (32). Analysis of feature category distributions further demonstrated that patients without LNM, with negative family history, and with low histological grade exhibited similar radiomic profiles across metastatic sites, indicating a more homogeneous and potentially less aggressive disease pattern (33). Conversely, the presence of LNM and high histological grade was strongly associated with greater heterogeneity and higher prevalence of visceral metastases. This pattern reinforces the link between classical clinicopathological markers of aggressiveness and distinct radiomic phenotypes predisposing to widespread dissemination. Notably, the observed association between positive family history and preferential bone metastasis suggests a possible hereditary predisposition not only to breast cancer development but also to metastatic tropism, warranting further exploration through genomic profiling. Collectively, these findings underscore the powerful role of radiomics in delineating the complex landscape of metastatic breast cancer. Integrating quantitative imaging biomarkers with established clinical variables—such as lymph node status, histological grade, and family history—enables more comprehensive and individualized prognostic assessment. This multi-dimensional approach holds promise for advancing precision oncology by improving metastasis risk stratification and providing a non-invasive basis for personalized treatment strategies.

The primary strength of this study lies in the use of real-world clinical imaging and pathological data, ensuring its relevance to actual clinical practice. This work represents substantial progress in precision oncology, leveraging machine learning and survival analysis for developing and validating individualized predictions for recurrence types and metastatic progression in breast cancer. The proposed models demonstrated high discriminatory performance and robustness, underscoring their potential to enhance clinical decision-making. However, several limitations should be acknowledged. First, the observed recurrence rate in this study was lower than the global average, possibly due to advancements in therapeutic interventions that have improved 5-year survival rates in recent years (34) and to selection bias stemming from cohorts based in resource-rich medical centers such as Shanghai. Second, although we employed inter-observer reproducibility analysis, test-retest stability assessment, and ComBat based cross center harmonization to enhance radiomic robustness, these approaches were implemented on retrospective data. Future prospective multi-center studies with standardized imaging protocols are warranted to further validate the generalizability of our radiomic signature. Third, The median follow-up duration for the cohort was 68.4 months. While this timeframe is suitable for assessing early-to-intermediate recurrence risk, it may be insufficient to capture very late recurrence events, particularly in hormone receptor-positive patients (35–38). Therefore, future studies with extended (>10-year) follow-up are warranted to validate the long-term prognostic performance of our model.

## 5 Conclusions

This study demonstrated that the combined radiomic and clinical signature tool effectively stratified patients into distinct recurrence-free survival (RFS) risk groups, outperforming conventional prognostic models. The clinical-radiomic nomogram exhibited a stronger correlation with RFS outcomes than traditional approaches, providing clinicians with an evidence-based framework for tailoring personalized therapeutic regimens for short- to medium term breast cancer patients. The radiomic score and Ki-67 index effectively differentiated between local recurrence, bone metastasis, and distant metastasis. However, further calibration and validation through high-quality prospective studies are required to confirm and refine these findings.

## Data Availability

The raw data supporting the conclusions of this article will be made available by the authors, without undue reservation.
