# Supplementary material for: Exploring the recurrence and metastasis of breast invasive ductal carcinoma based on machine learning and survival analysis
Source: Front Oncol. 2026 Mar 13;16:1734379. doi: 10.3389/fonc.2026.1734379 (PMC13021447; doi:10.3389/fonc.2026.1734379)
Supplement: Supplementary file 1 [file DataSheet1.docx]

Supplementary Material

# Supplementary Data

The hyperparameter search space for key algorithms included:

LGBM/XGBoost: max_depth [3, 5, 7], n_estimators [100, 200], learning_rate [0.01, 0.05, 0.1].

RF: n_estimators [100, 200, 300], max_depth [5, 10, None], min_samples_split [2, 5].

SVM: C [0.1, 1, 10], gamma [‘scale’, ‘auto’, 0.001, 0.01].

KNN: n_neighbors [3, 5, 7, 9], weights [‘uniform’, ‘distance’]

NN: A multilayer perceptron was tuned for hidden_layer_sizes [(50,), (100,), (50,50)], alpha (L2 penalty) [0.0001, 0.001], and learning_rate_init [0.001, 0.01].)

# Supplementary Figure


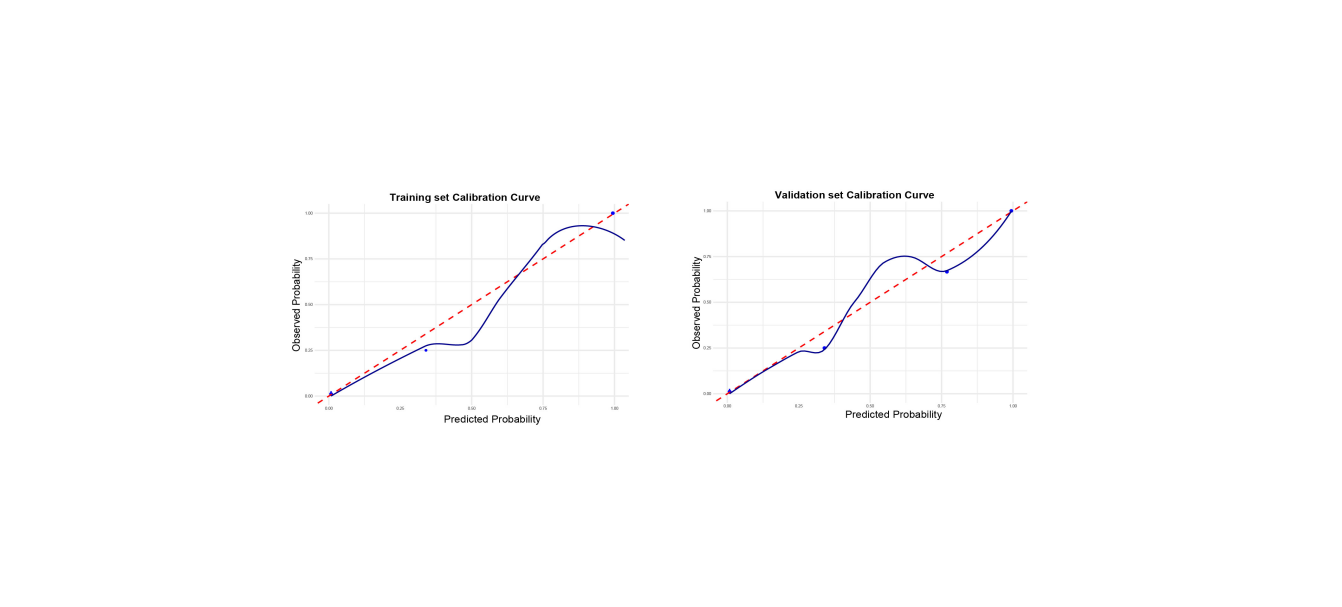


**Figure S1.** Calibration curves of the clinical-radiomic nomogram for predicting 5-year recurrence-free survival (RFS). （S1A）Calibration curve in the training cohort. Each point represents the predicted versus observed 5-year recurrence-free survival (RFS) rate within a risk group. The fitted line demonstrates high agreement between predictions and observations (calibration slope: 0.96; Hosmer-Lemeshow test, *p* = 0.32).（SIB）Calibration curve in the validation cohort. The model maintained good calibration in the external validation set (calibration slope: 0.92; Hosmer-Lemeshow test, *p* = 0.28).
